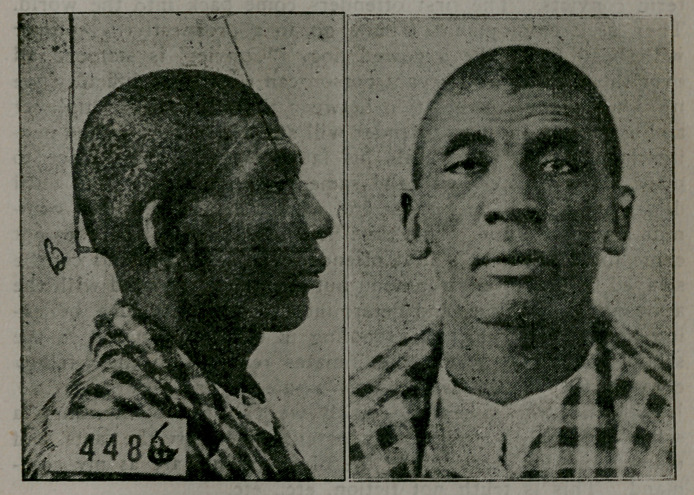# Some Anomalies of the Stigmata of Degeneracy

**Published:** 1907-11

**Authors:** Marc Ray Hughes

**Affiliations:** Professor of Mental and Nervous Diseases, Barnes University (Medical Department)—Professor of Criminal Anthropology, Benton College of Law—Associate Editor Alienist and Neurologist, St. Louis, Missouri


### SOME ANOMALIES OF THE STIGMATA OF DEGENERACY.

By MARC RAY HUGHES, M.D.,
Professor of Mental and Nervous Diseases, Barnes University (Medical Department)—Professor of Criminal Anthropology, Benton College of Law—Associate Editor Alienist and Neurologist, St. Louis, Missouri.

Mr. President, Ladies and Gentlemen:—

In broaching the subject of Criminal Anthropology, or the study of the criminal being, we have many skeins to unravel and many twisted and bifurcated paths of a dark and dismal labyrinth to explore, where obstacles meet us at every turn, obstacles thrown and placed in our path by a people who have a misconception of the terms stigmata and degeneration. When we think we have reached the last path in the great labyrinth that leads to success, where at the end of this darkened lane we can see the flickering light of the lamp of knowledge shedding its fair light in the dim distance, we imagine we are at our journey's end and success has crowned our effort, but alas, there just in front of our way in this dark path a shadow is cast and the rays of the lamp reacheth not a deep and yawning abyss, ready to engulf our efforts and mock at us in doleful echoes as we are hurled helplessly down to be dashed against its jagged sides, thrown from side to side until finally we rest on a ledge and untiringly begin once more our tedious ascent to the cherished goal. The public, swayed by the misconception of the subject of degeneracy, lay in our paths the obstacles and it is the deep and yawning chasm into which we seem helplessly to fall, but come forth again in our untiring efforts to benefit criminal beings and place them on a fair footing in the other world. It is the unknowing, unreasoning, unthinking masses, people whose brains are not capable of original reason that are led by a leader of equal mental stabiit yand force, that are responsible for the condition of the country so far as crime life is concerned. There are some, however, who deserve applause; though they are not of the multitude, they are of the class humane in nature who are assisting the under dog to a plane where it will be possible for him to earn an honest living and be of use to his fellow beings. Prisoners are not handled as though they were flesh and blood, but as some inanimate automatons, with an absolute conception of right and wrong as in a normal person. They are arrested by ignorant police and beaten into subjection and those blows upon the brain of an unstable neuropath or psycopath serve only to render the unfortunate human a more complete mental wreck and a burden on society for life. A man or woman lying in the doorway or on the sidewalk in down-town district or that of the tenderloin, supposedly a "drunk," by this mental giant, the policeman, is kicked, cuffed and beaten, when in many cases the unfortunate is an epileptic or has some other brain lesion and the roughness by which he is handled by the "strong arm of the law" renders him an object of pity whose place of refuge is the hospital and not the jail, to be brought before a magistrate to answer to a charge for a crime uncommitted.

If malformations like blows upon the head and toxines in the blood may modify character, we should look to find, as we do, variations of character in disease due to individualities of brain structure, both hereditary and acquired. Certain criminal acts are as much the result of diseased brains as other symptoms are the concomitants of other maladies. Malformation within the brain structure gives rise to these divisions, the insanities, criminal acts and other gross brain diseases, any one of which might run into the other, be a part of it or be dependent upon it; for instance, to begin with, given a brain lesion, epilepsy and crime the resultants. Those familiar with this branch of science will readily see the philosophy of the foregoing statement. I mention epilepsy as it is well known. When it has developed far enough to involve to a certain degree the psychic centres, the resultant oftentimes is mania of homicidal character, and homicide is crime.

Insanity is curable in many forms, so also is crime in many individuals. To discuss the methods that would tend to minimize criminal acts would take volumes, but I must mention in passing that the indeterminate sentence is one of the most important. I cannot do better than quote from my much esteemed friend and co-worker, William A. Hunter, late warden of the Anamosa State Penitentiary who, I regret to say, was taken away before the fruits of his energy had fully ripened, and whose death has been a loss to humanity in general. Together we had often discussed the good of the indeterminate sentence, and it is largely due to his efforts that some of the States now have that form of commitment.

In his report as chairman of the prison committee, he says:

"I must not enter into any exhaustive discussion of the Indeterminate Sentence, but briefly so far as it relates to prison discipline. The supreme and unanswerable argument for the indeterminate sentence is that it furnishes the criminal with the highest incentive for making character and preserving order, while it furnishes the management the broadest opportunity to induce self-control on the part of the criminal. Under this method the court says to him, 'I commit you to prison until you are fitted to become a citizen.' The Warden says to him, 'You are here and here to remain until you are fitted to take your place once more in the ranks of society. That day so longed for by you is to be fixed by no court, set by no governor, and to be moved by no influence, but it will come near or far, determined only by your own volition. Your future is in your own hands.' The criminal says to himself, 'Here I am —for how long? The law says that my friends are helpless to shorten my term and no appeal to the Executive can abate my sentence. Sullen looks, stubborn manners, rebellious conduct will only move the date farther down the calendar. There is only one thing which can influence the setting of that red letter day, and that is my own conduct.' You have enlisted his co-operation. All the internal man goes out to meet your external effort. What co-operation and profit-sharing is to the factory in promoting industry, order and production, the indeterminate sentence is to the prison. No greater crime against society could be committed than to turn loose a man who has been adjudged unfit to be at liberty, no matter whether he has been in prison for one year or for life. No greater injury could be done the man himself than to give him the hope that he can be released until he has squared his conduct with the moral law. Let the star of hope be inexorably kept shining over the pathway of virtue, but let it be as inexorably blotted out over the pathway of vice.

"A system of marks which classifies prisoners into grades, combined with the parole and indeterminate sentence enables you to teach the criminal the most valuable lesson for citizenship that he is likely to learn and that is, that it pays to do right—thus creating a powerful leverage for the regulation of human conduct. In the world from which he came the proverb that honesty is the best policy is at a discount. We must, in all fairness, admit that he is not to be severely blamed for this estimate of the economic value of righteousness. In the short run (and the criminal always lives in the short run) he has observed that trickery, dishonesty and thievery pay pretty good dividends and he comes to the conclusion that dishonesty is the best policy and that the Ten Commandments are out of date—who shall deny him the logic of the situation? He does not know that in the long run justice is always swifter than the feet of wrong and that history holds no criminal of yesterday who is not in the grip of remorse and reprobation to-day. He does not live in the long run—but from hand to mouth. Now he must learn that goodness pays, and that honesty is a good investment.

"Under the indeterminate system he sees his credit and debit marks piling up and sees the day of his emancipation shifting back and forth on the calendar, rising and falling in the gauge, registering accurately his progress in vice or virtue. The lesson that it pays to do right, the hardest of all lessons to teach children and harder still to teach men, is written before him night and day. It is inexorable, irrevocable. Admitting that often the sleekest criminal, the shrewdest and perhaps even the greatest will escape through the meshes of the indeterminate sentence, can it be denied that it quite often happens under the present regime? But even though the Indeterminate Sentence be imperfect we contend that it is still infinitely better for the criminal, for under this system he follows the path of virtue, sobriety, law and good order; he wears himself into a certain rut, the path he treads becomes a habit to him and habits are not thrown off in a day or in a month. The habit of observing good order and looking upon right as having a real and practical value cannot be easily shaken off, it is to have a grip on him which it never had before. Constant repetition cannot help making a habit, which, though it may be thrown off, to be sure, is also subject to the responsibility of becoming automatic. We have just as good a right to consult our hopes as our fears and to presume upon the good as upon the bad.

"If outside influence touches the indeterminate sentence plan with as much as its little finger, the whole scheme is lost, but we must admit that influence (political or otherwise) could not interfere more than it does under the definite sentence plan. If prisons are to do their required work in making men and creating character, the State should employ the very best penologists possible regardless of expense, give them an absolute, free hand and allow neither friends nor politicians, nor executive clemency to interfere, or the whole system is demoralized and the fruits of true discipline lost."

There must be two great divisions of crime, just as in lesions of the brain, namely, organic and inorganic or functional, the inorganic representing the volitional criminal, whether inherited or acquired, the organic representing the habitual moral imbecile, incorrigible and insane criminal; therefore, on the one hand certain forms of criminals are as incurable as certain other forms of mental disease. To say that a child born of criminal parents must be a criminal is preposterous—environment and discipline will dispel the inherited predisposition to crime, just as proper management will eradicate the predisposition to further unstableness that a child inherits from neuropathic or psycopathic parents. Given a child born of criminal parents. Take him from their influence and from criminal environment, direct his thoughts in the proper channels, the brain will begin to develop normally, he will acquire the habits of his environment and a normal being will be the result. The longer the criminal infant remains and is allowed to grow in an atmosphere of crime, the more imperfect becomes the brain formation and the harder it is to arrest his criminal tendencies. If allowed to run on, he finally becomes an instinctive criminal. The angles of the face and head show the equivalent of brain strength. In making anthropometric measurements, race idiosyncracies must be taken into consideration. Those races that have an index separate to themselves, such as the Indians, negroes, etc., and those whose indices must not be classified as belonging to the anomalies when it falls below or above the scale as in comparison with the anomalies of others showing other stigmata.

To give all of the anomalies of the stigmata of degeneracy would be going too much into detail, but suffice it to say that the measurements of the head and face are the first in importance of all the anthropometric measurements, then the eyes, ears, teeth, hard palate, skin and other anomalies of the general organism, too numerous to mention here. Every author and worker in criminal anthropology knows that it takes a number of anomalies to form the stigmata of degeneracy. Many people who are not familiar with the anthropology of the criminal or insane variety are prone to say that everyone is a criminal according to the anthropologist, but some day they will recognize the fact that no normal individual has a whole series of anomalies of the stigmata of degeneracy, as are found among the insane and criminal. One other point in favor of the indeterminate sentence if it ever supplants capital punishment, and that is the legal murder of those unfortunates who are not mentally able to control their impulses. Dr. Havelock Ellis says in his treatise on Anthropology, "The Criminal," "A very large number of crimes are committed by persons who are impelled by delusions or who have before the commission of the crime been in a condition of mental alienation. Nearly a hundred persons every year in this country (England) are sent to prison to be found insane on admission. The hanging of persons who are afterwards generally regarded as insane has been and is still, frequently carried on. In Germany Dr. Richter has shown that out of 144 lunatics who were, as was afterwards shown at the date of crimes in the highest degree insane, only 38 were recognized as insane by the judge; i. e., 106 madmen were on account of their madness, condemned to severe punishment. Out of 100 insane persons brought to the bar of justice, only 26 to 28 are recognized as insane, (vide infra) Sander & Richter (Die Beziehungen Zwischen Geistesstoerung und Verbrechen.)

If criminals were examined as to their sanity by anthropometric measurements alone, before they were brought before the bar, that grave mistake would not be made. A judge's power should rest at commitment, and as to duration, that is beyond him and should be left wholly in the hands of officers of the penitentiary, just as the commitment is, in reference to the insane; the judge can commit to an asylum, but he cannot determine the length of time or duration of the commitment. The two photographs speak for themselves, inmates of the Animosa Penitentiary. Have they not the marks of degeneracy? Judge for yourself. I have drawn the two facial angles so that you may see an easy method of determining the facial angles of degenerates. A simple measurement, and with a little knowledge of geometry, we are enabled to ascertain correctly their deficiencies. The angle is measured and worked by this formula:

$$\text{Cosine } C = \frac{\text{Side adjacent}}{\text{Hypotenuse}}$$

You have given in figure 4486, Hypotenuse and Side adjacent to find the degree of the angle C. Supposing the line B C measured 15 inches and the line A C measured 10 inches, then your angle worked out would read:

$$\text{Cos } C = \frac{\text{Angle } A}{H} = \frac{10}{15} = 66666$$

which is the Cos. C.

and the angle looked for would be 48 degrees, 48 minutes. If the other angle is wanted, the formula is reversed. All of the anomalies have an index and can be worked out and proven. The figure marked 4667 shows method of drawing for ascertaining both facial angles as are worked by the formulae above given. To give the methods of measuring anthropometrically, the other anomalies must be deferred to a future time, but in conclusion let me add the work of an anthropologist is that of befriending the criminal, and removing the conditions that have placed him or her in their present position, and elevating them to a place where they will be enabled to mingle once more with society, and given a chance to at least earn a living. "Once a criminal always a criminal," is the everlasting cry of the people who imagine themselves above every one else, and cultured to the highest degree, because they have enough to live on, and possibly some more, and do not think others less fortunate than themselves have a right to live, much less walk the streets in freedom. They are selfish to the very core, and think not of any one but themselves, and to hire even as a servant to do the most menial labor would be to their little minds a thing preposterous. If these pseudo-wise ones were as wise as they think, they would discharge from their employ some of their "trusted servants" and take in the one who frankly says when he applies for work, "I have just come from the penitentiary," because it is the novice and first offender that will own his guilt, but the third and fourth term criminal will deny it every time, though sooner or later it is found out. No one knows why each individual has committed his respective crime, until his story is told, and no one believes it when it is told, notwithstanding every word of it is true, simply because he has at one time been in the penitentiary. If in many instances the first offenders were given a show when released, the chances are that there would be no third or fourth term convicts. The first offenders come back into the world. They seek employment. They go to a prospective employer with head bent, like a cowed dog. "Convict" is stamped all over them, and an observant person can with little difficulty see it. During the conversation between the prospective employer and employee, the first offender will say he has been in the penitentiary. That word seals his fate. He goes from place to place to be greeted with the same response to his plea, until driven to it by sheer hunger and want the miserable soul commits a second crime. This time he is sentenced but he does not come forth again as a first offender who is supple and can be swayed by proper environment, but as one impregnated with the atmosphere of crime, and determined to do bigger jobs, believing he has had sufficient schooling in the art, acqurred in the school for crime, where the inmates rehearse to their fellow convicts the proper way to blow a safe, or break into a house, leaving no tracks behind. Shall we call the penitentiary a school for crime? Judge for yourself. These are the facts, and many more incidents in the life of a criminal are at my command. This is truth not fiction, etc., etc.

3872 Washington Ave.

## Figures and Tables

**Figure f1:**
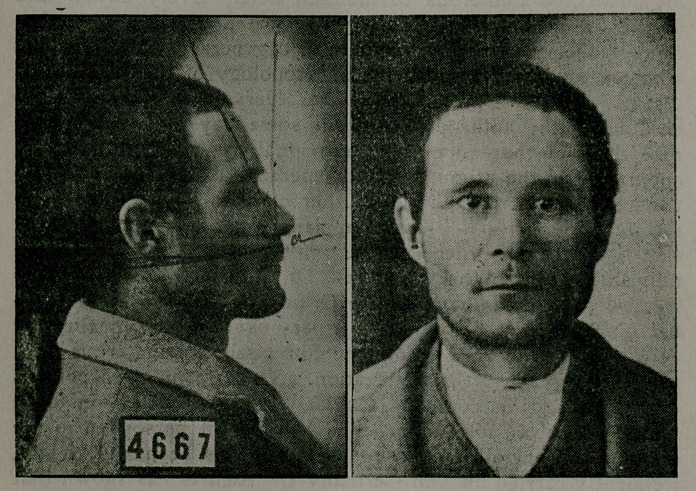


**Figure f2:**